# Diminazene aceturate attenuates hepatic ischemia/reperfusion injury in mice

**DOI:** 10.1038/s41598-022-21865-2

**Published:** 2022-10-28

**Authors:** So Hye Yoon, Hye Bin Kang, Jongwan Kim, Keunje Yoo, Sang Jun Han

**Affiliations:** 1 Department of Biotechnology, College of Fisheries Sciences, Pukyong National University, 45 Yongso-ro, Nam-gu, Busan, 48513 Republic of Korea; 2 Department of Medical Laboratory Science, Dong-Eui Institute of Technology, 54 Yangji-ro, Busanjin-gu, Busan, 47230 Republic of Korea; 3 Department of Environmental Engineering, Korea Maritime and Ocean University, Yeongdo-gu, Busan, 49112 Republic of Korea

**Keywords:** Drug discovery, Immunology, Medical research, Molecular medicine

## Abstract

Hepatic ischemia/reperfusion (I/R) injury is one of the leading causes of mortality following partial hepatectomy, liver transplantation, hypovolemic shock and trauma; however, effective therapeutic targets for the treatment of hepatic I/R injury are lacking. Recent studies have shown that diminazene aceturate (DIZE) has protective effects against inflammation, oxidative stress and cell death, which are the main pathogenetic mechanisms associated with hepatic I/R injury. However, the mechanistic effects DIZE exerts on hepatic I/R remain unknown. C57BL/6 male mice were pretreated with either 15 mg/kg DIZE or vehicle control (saline) and subjected to partial liver ischemia for 60 min. One day after induction of hepatic I/R, liver damage, inflammatory responses, oxidative stress and apoptosis were analyzed. By evaluating plasma alanine aminotransferase levels and histology, we found that DIZE treatment attenuated liver failure and was associated with a reduction in histologically-apparent liver damage. We also found that DIZE-treated mice had milder inflammatory responses, less reactive oxidative damage and less apoptosis following hepatic I/R compared to vehicle-treated mice. Taken together, our study demonstrates that DIZE protects against ischemic liver injury by attenuating inflammation and oxidative damage and may be a potential therapeutic agent for the prevention and treatment of ischemic liver failure.

## Introduction

Hepatic ischemia/reperfusion (I/R) injury is an unavoidable complication of liver surgeries such as liver resection and liver transplantation; it poses a major medical challenge and is associated with high morbidity and mortality, as patients subjected to partial hepatectomy, liver transplantation, and resuscitation after hypovolemic shock frequently suffer liver dysfunction due to I/R^1^. In addition, myocardial ischemia, stroke, abdominal trauma, and hemorrhagic shock can cause insufficient liver blood flow (ischemia), following restoration of blood flow (reperfusion), can lead to liver injury^2^. Various factors are involved in the pathophysiological process of hepatic I/R injury, including reactive oxidative damage to intracellular molecules, excessive inflammatory responses, apoptosis and necrosis of parenchymal cells such as hepatocytes, and intracellular organelle damage^3^. However, effective therapeutic targets for the clinical management of hepatic I/R are still lacking.

Diminazene aceturate (DIZE; 4-[2-(4-carbamimidoylphenyl) iminohy-drazinyl]benzenecarboximidamide) is an aromatic diamidine used extensively as a veterinary trypanocidal and babesiacidal agent^4^. Attention to this anti-parasitic drug has recently resurfaced because of its therapeutic potential in many human diseases, including pulmonary hypertension^5^, fulminant hepatitis^6^ and myocardial ischemia^7^. DIZE can regulate the host cellular and inflammatory responses by downregulating important intracellular signaling pathways that contribute to proinflammatory cytokine and chemokine production^8^ which is one of the major pathogenetic mechanisms of hepatic I/R injury. Mechanistically, it has been suggested that the anti-inflammatory effect of DIZE is mediated by the activation of angiotensin-converting enzyme 2 (ACE2), which converts angiotensin 2 (Ang II) to angiotensin 1–7 (Ang (1–7)), mainly in cardiovascular diseases^7,9^.

The renin-angiotensin system (RAS) is involved in vascular resistance, blood pressure regulation, sodium and water homeostasis and tissue remodeling following injury^10^. ACE2 attenuates an activated ACE/Ang II pathway by degrading Ang II and producing Ang (1–7). The relative balance between the negative ACE/Ang II and the beneficial ACE2/Ang (1–7) pathways appears to be a significant mediator of tissue injury^11^. Besides being an ACE2 activator, beneficial ACE2-independent mechanisms of action have also been attributed to DIZE in some pathological conditions^9^. Indeed, DIZE attenuates tissue injury in animal models of myocardial infarction^7^ and renal ischemia^12^. However, the role of DIZE in hepatic I/R injury remains unclear. In this study, we investigated the effect of DIZE on hepatic I/R injury and its therapeutic potential for the treatment and prevention of hepatic I/R-induced acute liver failure.

## Methods

### Animal preparation

Eight-week-old C57BL/6 male mice (weighing 22–26 g) were anesthetized intraperitoneally with pentobarbital sodium (50 mg/kg; Hanlim Pharma Co., Seoul, Korea) and subjected to 1 h of partial hepatic I/R injury or sham surgery as described previously^13^; all procedures were approved by the Institutional Animal Care and Use Committee of Pukyong National University. The study was conducted in accordance with the Guide for the Care and Use of Laboratory Animals published by the US National Institutes of Health (NIH Publication No. 85–23, revised 2011). The study was carried out in compliance with the ARRIVE guidelines. Sham-operated mice were subjected to laparotomy and identical liver manipulation without vascular occlusion. The mice were provided free access to water and standard chow. The animal models were divided into groups as follows: (1) Sham surgery group pre-treated with vehicle (saline); (2) sham surgery group pre-treated with DIZE (15 mg/kg); (3) hepatic I/R group pre-treated with vehicle (saline); (4) hepatic I/R group pre-treated with DIZE (15 mg/kg). The DIZE dose was chosen based on previously reported studies^6,12,14^. DIZE and vehicle were administered intraperitoneally at 48 h, 24 h and 1 h before surgery. The body temperature was maintained at 36.5–37 °C throughout all surgical procedures using a temperature-controlled heating device (FHC, Bowdoin, ME). Twenty-four hours after reperfusion, the liver tissues were collected; blood was also collected at 4 and 24 h after reperfusion. To investigate histological damage, oxidative damage, inflammation, and apoptosis, liver tissues were frozen in liquid nitrogen or fixed in neutral 10% formalin (Sigma-Aldrich, St. Louis, MO, USA).

### Measurement of hepatic injury after surgery

We measured plasma alanine aminotransferase (ALT) levels at 4 and 24 h post hepatic I/R. Serum was collected at 4 and 24 h following completion of hepatic I/R induction or sham surgery. Alanine transaminase (ALT) was measured using an alanine transaminase assay kit (Bioassay System, Hayward, CA, USA). To assess liver morphological damage, liver H&E sections were graded for I/R injury using the Suzuki score system^15^. In this classification, three liver injury indices are graded: sinusoidal congestion (0–4), cytoplasmic vacuolization (0–4), and parenchymal necrosis (0–4); absence of parenchymal necrosis, sinusoidal congestion, or cytoplasmic vacuolization were assigned a score of 0, whereas severe sinusoidal congestion, severe cytoplasmic vacuolization, and > 60% parenchymal necrosis were given a value of 4. Based on these three indices, each H&E sample was graded with a cumulative score (range of 0–12).

### Western blot analysis

Liver tissues were lysed using RIPA lysis buffer (150 mM NaCl, 1% triton-X 100, 0.5% sodium deoxycholate, 0.1% SDS, 50 mM Tris–HCl) supplemented with protease and phosphatase inhibitors (Sigma-Aldrich). Protein samples were separated by SDS-PAGE and transferred to a polyvinylidene difluoride (PVDF) membrane (GVS, Bologna, Italy). After blocking with 5% BSA or skim milk for 30 min, PVDF membranes were incubated overnight with primary antibodies against B-cell lymphoma 2 (Bcl-2, 1:2000, Cell Signaling Technology Danvers, MA), cleaved caspase-3 (1:2000, Cell Signaling Technology, Danvers, MA), Ly6G (1:2000, eBioscience, San Diego, CA ), 4-hydroxynonenal (4-HNE, 1:2000, Abcam, Cambridge, UK), NFκB p65 (1:2000, Cell Signaling Technology, Danvers, MA), IκBa (1:2000, Cell Signaling Technology, Danvers, MA) and GAPDH (1:10,000, Bioworld Technology, St. Louis Park, MN). The membranes were then incubated with HRP-labelled goat anti-mouse IgG (1:3000, BETHYL-Laboratories, Montgomery, TX) or goat anti-rabbit IgG (1:3000, BETHYL-Laboratories, Montgomery, TX) secondary antibodies for 1 h at room temperature. Total protein expression levels were normalized to GAPDH (Supplementary Figures). The intensity of each protein band was analyzed using the ImageJ v.1.53e software (NIH, Bethesda, MD, USA, https://imagej.nih.gov/ij).

### Immunohistochemical staining and TUNEL staining

Liver neutrophil infiltration and generation of 8-hydroxy-2-deoxyguanosine (8-OHdG) after hepatic IR injury were detected by immunohistochemistry using a rat anti-mouse Ly6G monoclonal antibody (1:100, eBioscience, San Diego, CA) and an anti-8-hydroxy-2-deoxyguanosine antibody (1:1000, Abcam, Cambridge, UK). Neutrophils were counted from randomly chosen 200× microscope image fields. The 8-OHdG intensity was measured in a randomly selected 200× microscope image field using the Fiji Image J v.1.53c software (NIH, Bethesda, MD, USA, https://fiji.sc). The terminal deoxynucleotidyl transferase-mediated dUTP-digoxigenin nick end labeling (TUNEL) assay was performed using a Deadend Fluorometric TUNEL System Kit (Promega, Madison, WI, USA), according to the manufacturer’s instructions. Nuclei with clear green staining were regarded as TUNEL-positive apoptotic cells. TUNEL-positive apoptotic cells were counted from randomly chosen 200X microscope image fields.

### Quantitative RT-PCR

Quantitative real-time PCR was performed to evaluate the expression of mRNAs encoding proinflammatory markers in the liver following surgery. Among the mRNAs assessed were tumor necrosis factor α (*TNF-α*), interleukin-1β (*IL-1β*), interleukin-6 (*IL-6*), keratinocyte chemoattractant (*KC*), monocyte chemoattractant protein-1 (*MCP-1*), and macrophage inflammatory protein-2 (*MIP-2*); the primers used are listed in Table 1. Total RNA was extracted from liver tissues using TRIzol (Ambion, Austin, TX). Using reverse-transcription PCR, extracted RNA from each sample was converted into cDNA using random primers. The levels of respective cDNAs were then assessed by quantitative PCR (Biorad, Hercules, CA, USA), using the FastStart Universal SYBR Green Master Mix (Sigma-Aldrich, St. Louis, MO, USA). To confirm equal RNA input, mRNA expression levels were normalized to GAPDH. Relative expression was calculated using the ΔΔCt method (Ct: threshold cycle), and specificity of the amplification was confirmed by melting curve analysis.

**Table 1:** Primers used in quantitative reverse transcription polymerase chain reactions to amplify mouse cDNAs based on published GenBank sequences.

Primers	Sequence (sense/antisense)	Annealing temp (°C)
mouse TNF-α	5′-TACTGAACTTCGGGGTGATTGGTCC-3′	65 °C
	5′-CAGCCTTGTCCCTTGAAGAGAACC-3′	
mouse MCP-1	5′-ACCTGCTGCTACTCATTCAC-3′	60 °C
	5′-TTGAGGTGGTTGTGGAAAAG-3′	
mouse MIP-2	5′-CCAAGGGTTGACTTCAAGAAC-3′	60 °C
	5′-AGCGAGGCACATCAGGTACG-3′	
mouse KC	5′-CAATGAGCTGCGCTGTCAGTG-3′	60 °C
	5′-CTTGGGGACACCTTTTAGCATC-3′	
mouse IL-6	5′-CCGGAGAGGAGACTTCACAG-3′	62°C
	5′-GGAAATTGGGGTAGGAAGGA-3′	
mouse IL-1β	5′-CTGAAAGCTCTCCACCTC-3′	56 °C
	5′-TGCTGATGTACCAGTTGGGG-3′	
GAPDH	5′-ACCACAGTCCATGCCATCAC-3′	65 °C
	5′-CACCACCCTGTTGCTGTAGCC-3′	

Annealing temperatures used for each primer are also provided.

### Measurement of angiotensin 2 and angiotensin (1–7)

Plasma and liver levels of angiotensin 2 and angiotensin (1–7) were measured 24 h following completion of hepatic I/R induction or sham surgery. Angiotensin 2 and angiotensin (1–7) levels were quantified using an angiotensin 2 ELISA kit (Cusabio, Houston, TX, USA) and angiotensin (1–7) ELISA Kit (Novus Biologicals, Columbus, OH, USA) respectively.

### Statistical analysis

Results are expressed as mean ± SEM. Statistical differences among groups were calculated using Student’s *t* test, the Mann–Whitney nonparametric test and one-way analysis of variance (ANOVA with plus Tukey’s post hoc multiple comparison test) was used for comparing each groups. Analysis of variance was measured using Origin 2021 v.9.8.0 200 (OriginLab, Northampton, MA, USA, https://www.originlab.com). Statistical significance was set at P < 0.05.

## Results

### DIZE pretreatment alleviates liver injury following hepatic I/R

**Figure 1:**
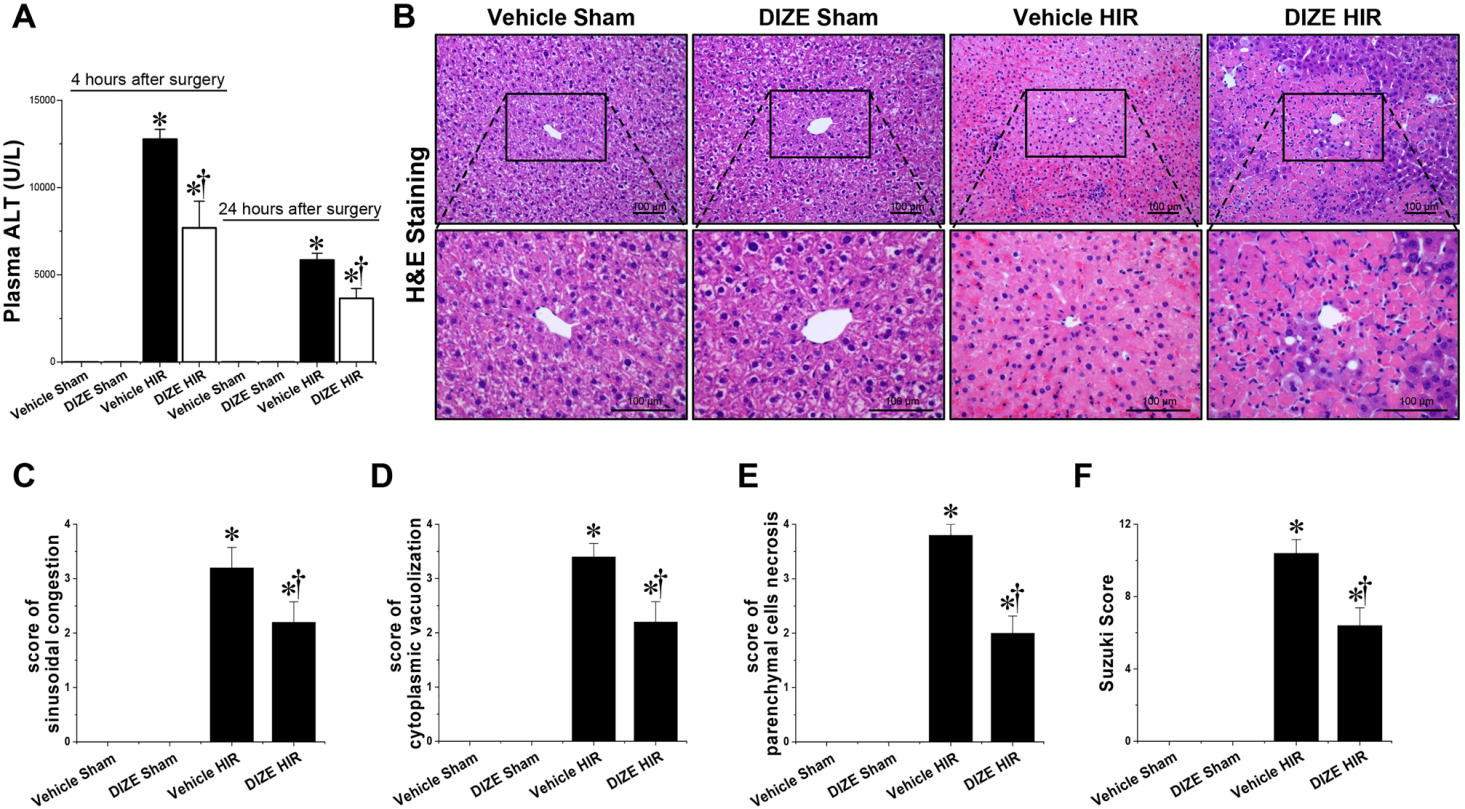
Effect of DIZE on liver injury following hepatic ischemia/reperfusion. C57BL/6 male mice were subjected to partial hepatic ischemia (HIR) induction for 1 h or sham surgery. Mice were randomized to receive treatment with either Diminazene aceturate (DIZE) or vehicle control (saline) before undergoing surgery, as described in “Methods”. (**A**) Four and twenty-four hours after surgeries, plasma ALT levels were measured. Liver sections were subjected to hematoxylin and eosin (H&E) staining (**B**, Upper panel: magnification X200; lower panel: magnification X400) and histological damages (**C**, **D**, **E**, and **F**) were analyzed using Suzuki’s criteria (sinusoidal congestion, parenchymal cell necrosis and cytoplasmic vacuolization). Results are expressed as mean ± SEM (n = 5). For statistical analysis, the one-way ANOVA plus Tukey’s post hoc multiple comparison test was used to detect significant changes in plasma ALT levels and the Mann–Whitney nonparametric test and *t* test were used to detect significant changes of Suzuki score with Origin 2021 v.9.8.0 200 (OriginLab, https://www.originlab.com) software. *P < 0.05 vs. Vehicle Sham, †P < 0.05 vs. Vehicle HIR.

First, we tested whether DIZE treatment protected against hepatic I/R injury. The levels of serum ALT were similar between vehicle-and DIZE-pretreated mice subjected to sham operation (Fig. 1A). Plasma ALT levels increased 4 and 24 h post hepatic I/R in both vehicle- and DIZE-pretreated mice. However, DIZE pretreatment was associated with a significantly lower increase in serum ALT, both at 4 and 24 h after hepatic I/R injury (Fig. 1A). Next, we analyzed liver H&E-stained slides for the degree of hepatocellular damage using the Suzuki’s criteria^15^. Vehicle-pretreated mice subjected to hepatic I/R injury had severe sinusoidal congestion, cytoplasmic vacuolization, and necrosis of hepatocytes, whereas DIZE pretreatment significantly alleviated hepatic IR-induced liver damage (Fig. 1B–F). We failed to detect necrosis or liver injury in the liver sections of sham-operated mice (Fig. 1B–F). Taken together, these results demonstrate that DIZE can attenuate hepatic IR-induced liver damage.

### DIZE pretreatment reduces apoptosis in the liver following hepatic I/R

**Figure 2:**
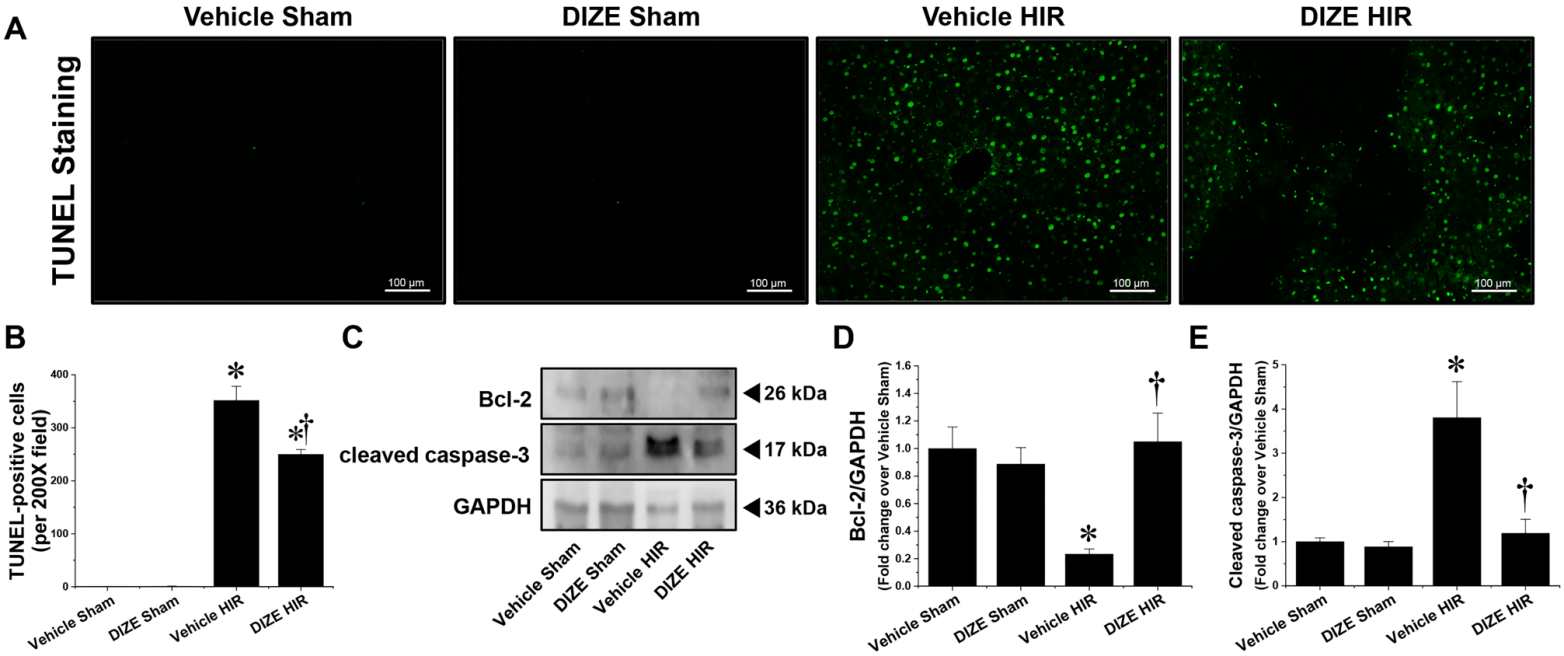
Effect of DIZE on apoptosis following hepatic ischemia/reperfusion. C57BL/6 male mice were subjected to partial hepatic ischemia (HIR) induction for 1 h or sham surgery. Mice were treated with either Diminazene aceturate (DIZE) or vehicle control (saline) before undergoing surgery, as described in “Methods”. Twenty-four hours after surgery, liver sections were subjected to TUNEL staining (**A**; magnification X200) and the TUNEL-positive cells were counted (**B**). (**C**) Whole liver lysates were subjected to western blotting using B-cell lymphoma 2 (Bcl-2) and cleaved caspase-3 antibodies. GAPDH was used as a loading control. (**D, E**) Band intensities were measured using the ImageJ v.1.53e software. Results are expressed as mean ± SEM (n = 5). For statistical analysis, one-way ANOVA with plus Tukey’s post hoc multiple comparison test were used to detect significant changes with Origin 2021 v.9.8.0 200 (OriginLab, https://www.originlab.com) software. *P < 0.05 vs. Vehicle Sham, †P < 0.05 vs. Vehicle HIR.

Apoptosis is the primary manifestation of hepatic I/R injury, and together with necrosis, is one of the main mechanisms of cell death during hepatic I/R^16^. Apoptotic cell death in liver tissues was determined by TUNEL staining, and expression of proteins involved in the apoptotic pathway such as Bcl-2 and cleaved caspase-3 was measured by western blotting. Figure 2A, B show representative TUNEL staining images indicating apoptosis and counts of TUNEL-positive cells in the livers of mice subjected to sham surgery or hepatic IR. The number of TUNEL-positive cells did not significantly differ between vehicle- and DIZE-pretreated mice subjected to sham surgery, and it increased in both groups following hepatic I/R injury. However, the increase in TUNEL-positive cells in the liver was lower in DIZE-pretreated mice compared to vehicle-pretreated control mice (Fig. 2A, B).

Consistent with the TUNEL assay data, western blot analysis revealed that the expression of the anti-apoptotic protein Bcl-2 decreased, whereas the cleaved (active) form of the pro-apoptotic caspase-3 increased following hepatic I/R injury (Fig. 2C–E); however, DIZE pretreatment attenuated these changes. These results indicate that DIZE pretreatment reduced intrahepatic apoptosis induced by hepatic I/R injury.

### DIZE pretreatment alleviates hepatic inflammation following hepatic I/R

**Figure 3:**
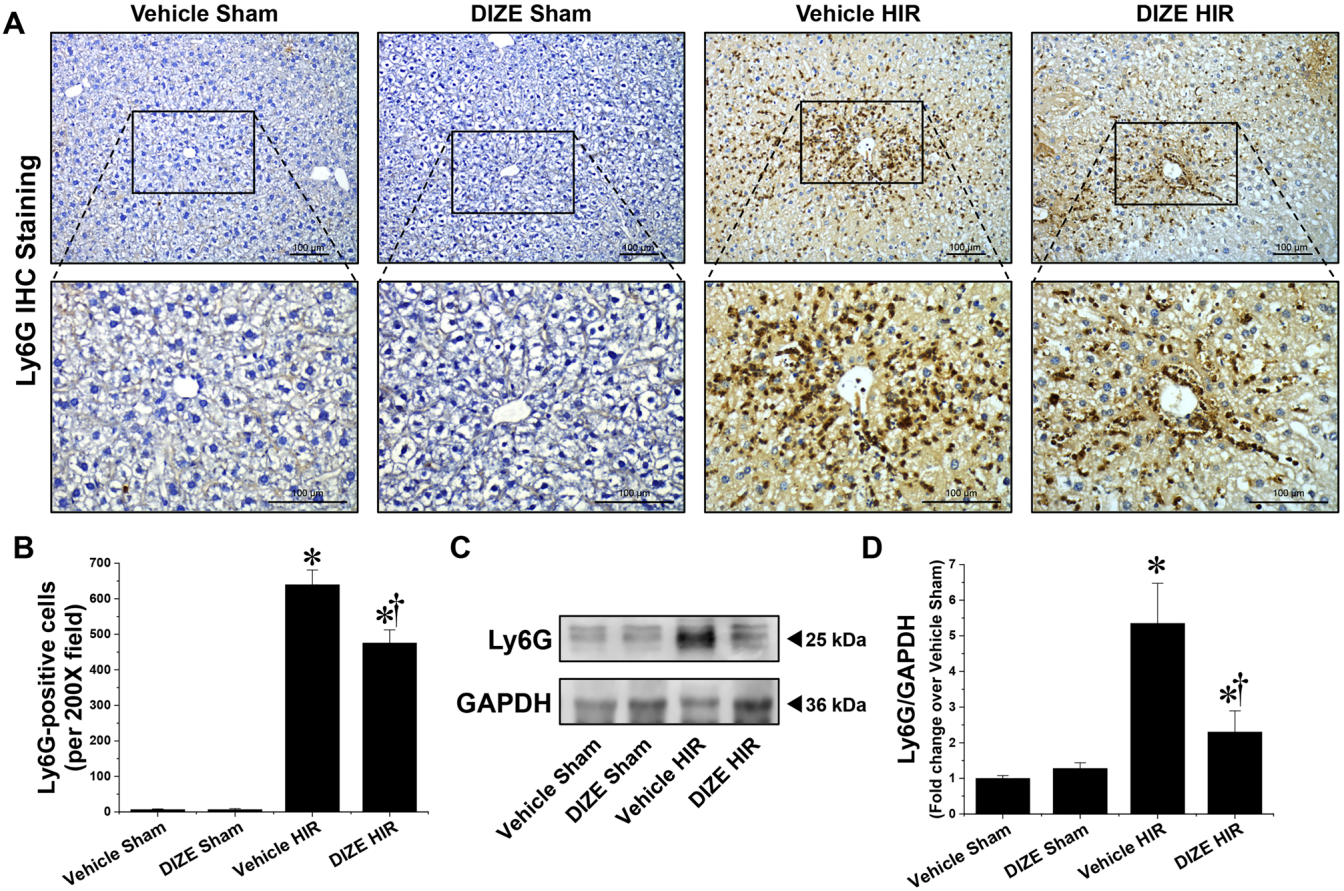
Effect of DIZE on neutrophil infiltration and Ly6G protein expression following hepatic ischemia/reperfusion. C57BL/6 male mice were subjected to partial hepatic ischemia (HIR) induction for 1 h or sham surgery. Mice were treated with either Diminazene aceturate (DIZE) or vehicle control (saline) before undergoing surgery, as described in “Methods”. Twenty-four hours after surgery, liver sections were subjected to immunohistochemistry (IHC) staining using an antibody against Ly6G, a neutrophil marker (**A**, Upper panel: magnification X200; lower panel: magnification X400), and Ly6G-positive cells were counted (**B**). (**C**) Whole liver lysates were subjected to western blot analysis using an anti-Ly6G antibody. GAPDH was used as a loading control. (**D**) Band intensities were measured using the ImageJ v.1.53e software. Results are expressed as mean ± SEM (n = 5). For statistical analysis, one-way ANOVA with plus Tukey’s post hoc multiple comparison test were used to detect significant changes with Origin 2021 v.9.8.0 200 (OriginLab, https://www.originlab.com) software. *P < 0.05 vs. Vehicle Sham, †P < 0.05 vs. Vehicle HIR.

Figure 3A, B show representative immunohistochemistry images and counts of Ly6G-positive cells in the liver of vehicle- and DIZE-pretreated mice subjected to sham surgery or hepatic I/R injury. Neutrophil infiltration in the liver increased as a result of hepatic I/R injury; however, DIZE pretreatment was associated with a lower increase. Similarly, the total protein expression of Ly6G evaluated by western blotting increased as a result of hepatic I/R injury, but this increase was dampened by DIZE pretreatment (Fig. 3C, D).

**Figure 4:**
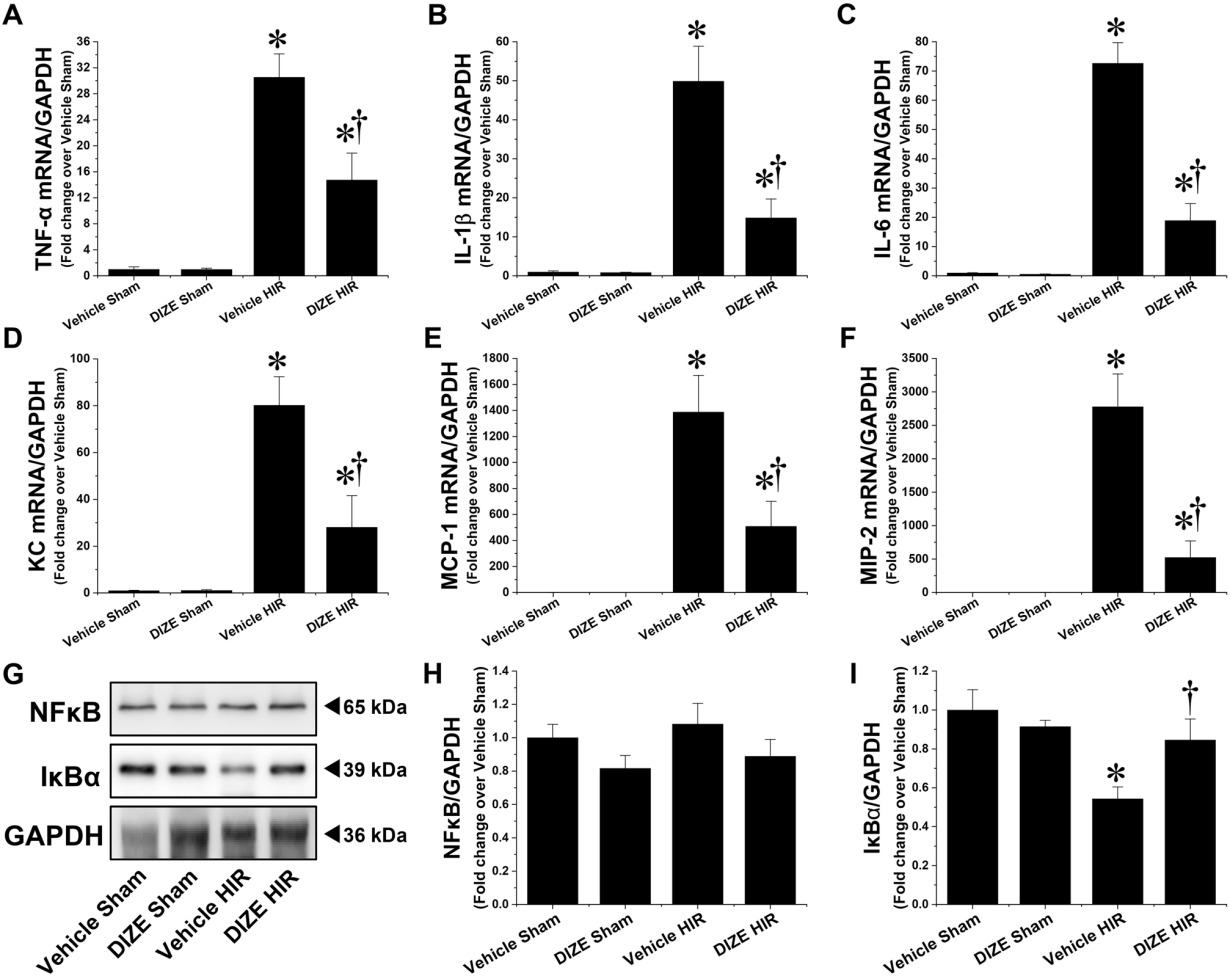
Effect of DIZE on hepatic inflammation following hepatic ischemia/reperfusion. C57BL/6 male mice were subjected to partial hepatic ischemia (HIR) induction for 1 h or sham surgery. Mice were treated with either Diminazene aceturate (DIZE) or vehicle control (saline) before undergoing surgery, as described in “Methods”. Fold-increases in the liver expression of pro-inflammatory mRNAs [tumor necrosis factor α (*TNF-α*, **A**), interleukin 1β (*IL-1β*, **B**), interleukin 6 (*IL-6*, **C**), keratinocyte chemoattractant (*KC*, **D**), monocyte chemoattractant protein-1 (*MCP-1*, **E**) and macrophage inflammatory protein-2 (*MIP-2*, **F**)] were assessed using quantitative RT-PCR 24 h after sham surgery or HIR induction; expression of GAPDH was used as reference control. (**G**) Liver samples were subjected to western blot analysis using anti-NFκB and -IκBα antibodies. (**H, I**) Band intensities were measured using the ImageJ v.1.53e software. Results are expressed as mean ± SEM (n = 5). For statistical analysis, one-way ANOVA with plus Tukey’s post hoc multiple comparison test were used to detect significant changes with Origin 2021 v.9.8.0 200 (OriginLab, https://www.originlab.com) software. *P < 0.05 vs. Vehicle Sham. †P < 0.05 vs. Vehicle HIR.

Figure 4A–F shows fold-increases in the expression of proinflammatory mRNAs in the liver, following hepatic I/R injury; the expression of the indicated mRNAs is normalized to that of GAPDH. Compared to sham mice, hepatic I/R injured mice showed elevated mRNA levels of proinflammatory cytokines and chemokines, including TNF-α, IL-1β, IL-6, KC, MCP-1 and MIP-2. Interestingly, upregulation of all these mRNAs was prevented by DIZE pretreatment, indicating that DIZE reduces the pro-inflammatory response induced by hepatic I/R injury.

Since NFκB is a well-known transcription factor that promotes the synthesis of pro-inflammatory cytokines and chemokines during hepatic I/R injury^17^, we examined whether DIZE pretreatment attenuated NFκB signaling activation following hepatic I/R injury. Protein expression of IκBα and NFκB p65 were confirmed by western blotting. As shown in Fig. 4G–I, expression levels of IκBα were significantly reduced in the liver of vehicle-treated mice following hepatic I/R injury, and DIZE pretreatment significantly attenuated this decrease. In contrast, total NFκB protein expression was not significantly altered by hepatic I/R injury and DIZE pretreatment. However, the decrease in IκBα protein expression was significantly attenuated by DIZE pretreatment following hepatic I/R injury. Although the exact regulation of NFκB nuclear translocation by DIZE remains to be defined, these results suggest that DIZE pretreatment attenuates the NFκB signaling pathway by preventing IκBα degradation following hepatic I/R injury.

### DIZE pretreatment decreases oxidative stress following hepatic I/R

**Figure 5:**
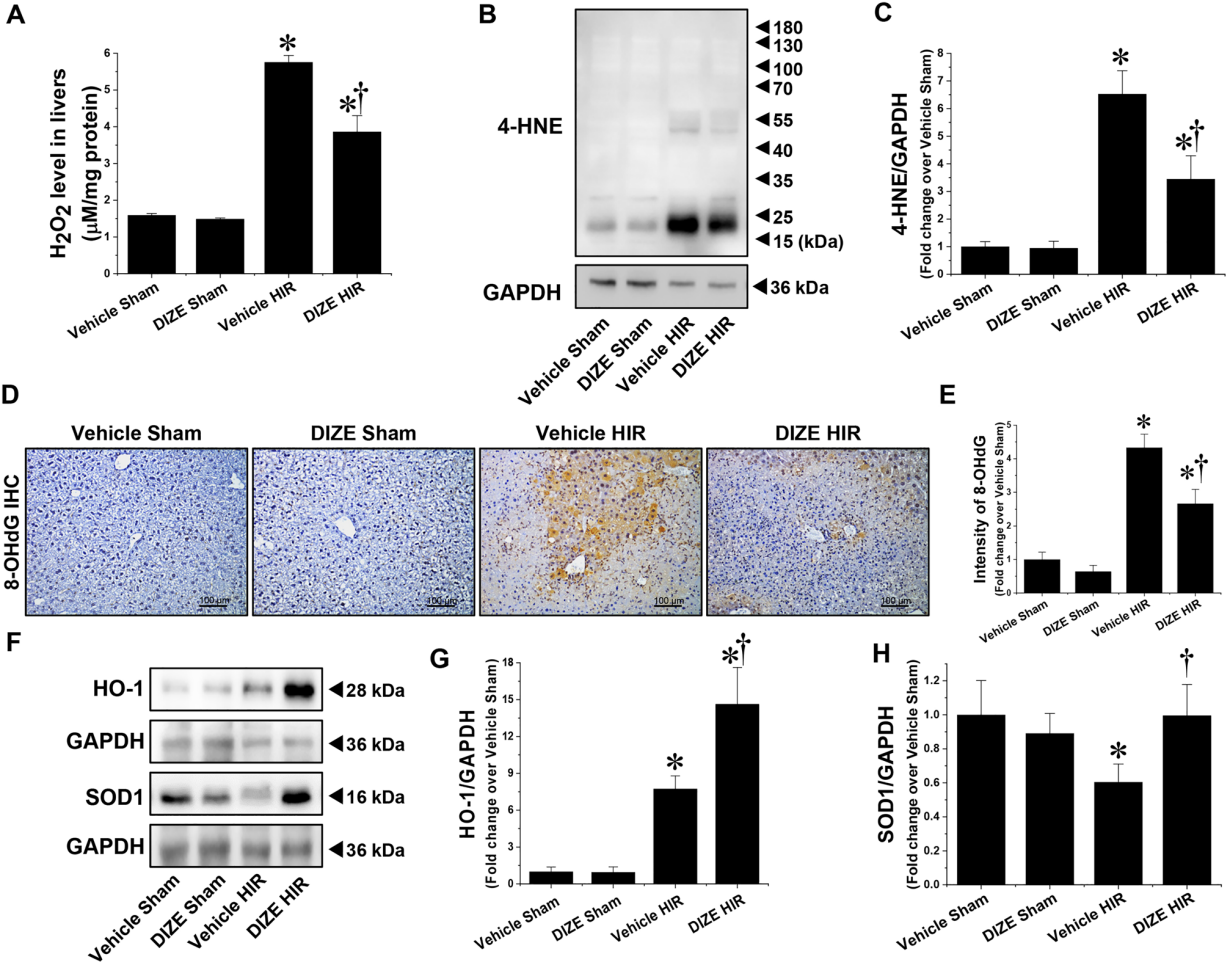
Effect of DIZE on oxidative stress following hepatic ischemia/reperfusion. C57BL/6 male mice were subjected to partial hepatic ischemia (HIR) induction for 1 h or sham surgery. Mice were treated either with Diminazene aceturate (DIZE) or vehicle control (saline) before undergoing surgery, as described in “Methods”. (**A**) Levels of hydrogen peroxide in the liver were measured. (**B**) Whole liver lysates were subjected to western blot analysis using an anti-4-Hydroxynonenal (4-HNE) antibody. GAPDH was used as a loading control. (**C**) Band intensities were measured using the ImageJ software. Liver sections were subjected to immunohistochemistry (IHC) staining using an anti-8-hydroxy-2′-deoxyguanosine (8-OHdG) antibody (**D**; magnification X200). (**E**) The intensity of 8-OHdG staining was measured using Fiji imageJ v.1.53c software. (**F**) Whole liver lysates were subjected to western blot analysis using anti-Heme oxygenase 1 (HO-1) and Superoxide dismutase 1 (SOD1) antibodies. GAPDH was used as a loading control. (**G, H**) Band intensities were measured using the ImageJ v.1.53e software. Results are expressed as mean ± SEM (n = 5). For statistical analysis, one-way ANOVA with plus Tukey’s post hoc multiple comparison test were used to detect significant changes with Origin 2021 v.9.8.0 200 (OriginLab, https://www.originlab.com) software. *P < 0.05 vs. Vehicle Sham, †P < 0.05 vs. Vehicle HIR.

The redox balance is disrupted during hepatic I/R, thereby leading to an accumulation of reactive oxygen species (ROS) and oxidative damage to macromolecules, including proteins, lipids, and nucleic acids; this is one of the major pathogenic mechanisms underlying hepatic I/R injury^3^. We therefore analyzed H_2_O_2_ levels, lipid peroxidation, DNA oxidation, and protein expression of antioxidant enzymes. H_2_O_2_ and 4-HNE levels, both indexes of lipid peroxidation, were significantly increased in the liver of mice following hepatic I/R injury, and this increase was attenuated by DIZE pretreatment (Fig. 5A–C). Similarly, the intensity of 8-OHdG, a marker of oxidative stress, significantly increased following liver I/R injury and DIZE pretreatment attenuated this increase (Fig. 5D, E). Furthermore, expression of heme oxygenase 1 (HO-1), an Nrf2-regulated survival factor that plays a critical role in preventing inflammation and oxidative stress^18,19^ was increased in DIZE-pretreated animals following hepatic I/R injury (Fig. 5F, G). Finally, expression of superoxide dismutase 1 (SOD1), an antioxidant against superoxide, was decreased following hepatic I/R injury, but this decrease was prevented by DIZE pretreatment (Fig. 5F, H). There were no significant differences in H_2_O_2_ level, lipid peroxidation, and protein expression of HO-1 and SOD1 between sham-operated vehicle- and DIZE-treated mice (Fig. 5).

### DIZE pretreatment increases ACE2 activity in plasma following hepatic I/R

**Figure 6:**
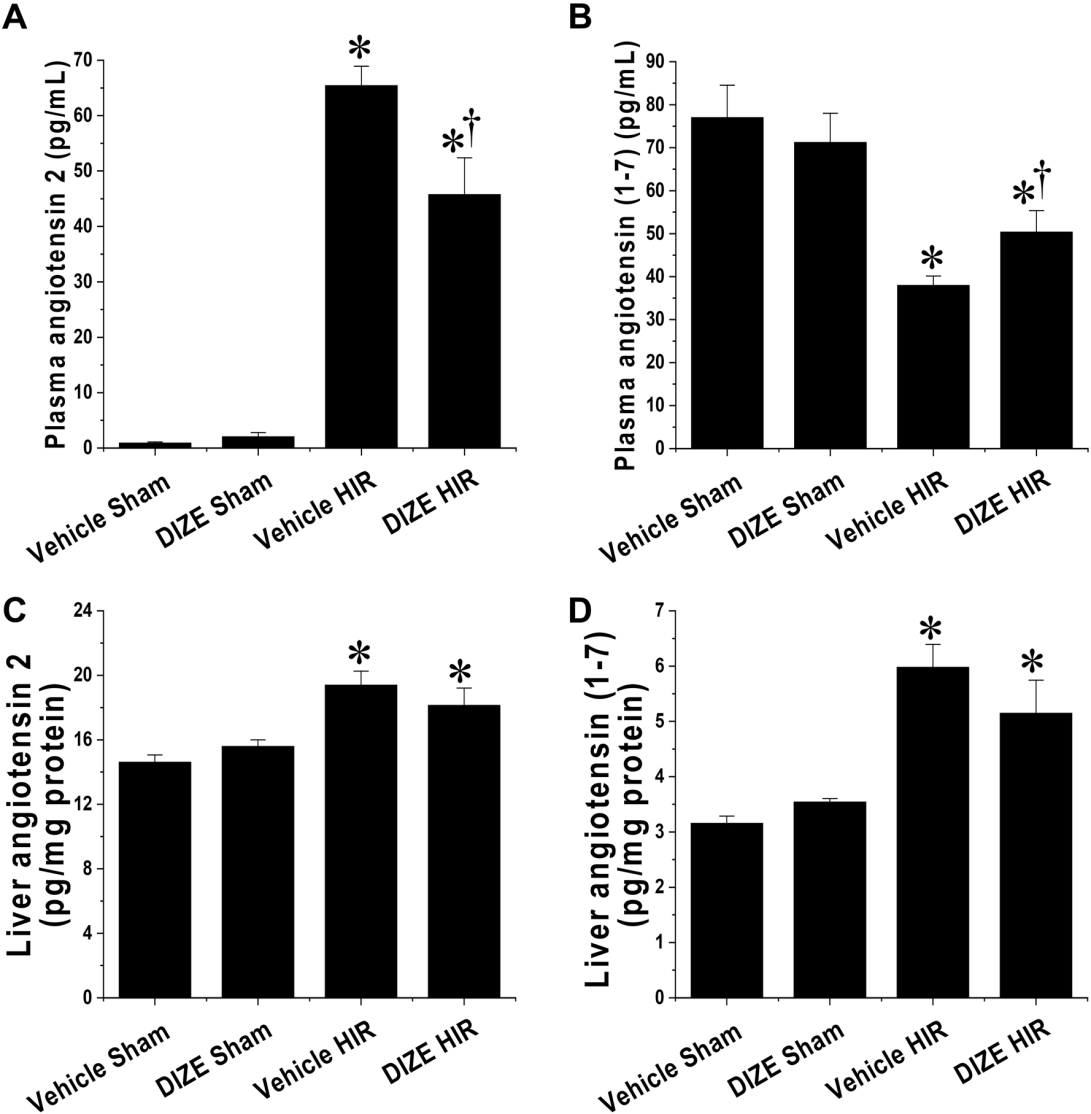
Effect of DIZE on angiotensin 2 and angiotensin (1–7) levels in plasma and liver, following hepatic ischemia/reperfusion. C57BL/6 male mice were subjected to partial hepatic ischemia (HIR) induction for 1 h or sham surgery. Mice were treated either with Diminazene aceturate (DIZE) or vehicle control (saline) before undergoing surgery, as described in “Methods”. Twenty-four hours after surgery, angiotensin 2 and angiotensin (1–7) levels were measured in plasma and liver. Results are expressed as mean ± SEM (n = 5). For statistical analysis, one-way ANOVA with plus Tukey’s post hoc multiple comparison test were used to detect significant changes with Origin 2021 v.9.8.0 200 (OriginLab, https://www.originlab.com) software. *P < 0.05 vs. Vehicle Sham, †P < 0.05 vs. Vehicle HIR.

Lastly, we measured the protein levels of angiotensin (1–7) and angiotensin 2 in the plasma and liver tissues to determine whether DIZE pretreatment activated the ACE2/Ang (1–7)/Mas receptor pathway. We found that angiotensin 2 levels in plasma significantly increased, whereas angiotensin (1–7) levels in plasma decreased following hepatic I/R injury. However, DIZE pretreatment ameliorated the increase in plasma angiotensin 2 and the decrease in plasma angiotensin (1–7) after hepatic I/R injury (Fig. 6A, B). In liver tissues, DIZE pretreatment didn’t affect the changes of angiotensin 2 and angiotensin (1–7) protein levels after sham or hepatic I/R surgeries (Fig. 6C, D).

## Discussion

Here, we first describe the protective effect of DIZE against hepatic I/R injury. The plasma ALT levels and histological liver damage in the DIZE-pretreated hepatic I/R group were significantly lower compared to the vehicle-treated hepatic I/R group. In addition, DIZE pretreatment protected against apoptotic liver cell death, inflammation, and oxidative stress induced by hepatic I/R injury. Based on these results, treatment with DIZE could be considered as a novel therapeutic approach for preventing/treating hepatic I/R-induced liver failure.

Inflammatory responses significantly contribute to tissue damage that occurs during hepatic I/R. One of the major cell types involved in the inflammatory responses during hepatic I/R injury is Kupffer cells; these are activated in the early stage of hepatic I/R injury and release inflammatory cytokines such as TNF-α, IL-1β, IL-6, ROS, and proteases^20,21^. These cytokines induce the expression of neutrophil-attracting chemokines such as KC and MIP-2 and upregulate vascular adhesion molecules, thereby amplifying inflammation and oxidative stress^20^. Neutrophils are the first accumulating leukocytes in the liver during hepatic I/R injury and are known to be a major contributor to additional liver injury after reperfusion^22^. Reduced liver neutrophil infiltration and mRNA expression of inflammatory cytokines and chemokines (TNF-α, IL-1β, IL-6, KC, MCP-1, and MIP-2) in DIZE-pretreated mice may contribute to improved liver function after hepatic I/R injury. Furthermore, we observed that IκBα protein expression was significantly reduced in the liver of vehicle-treated mice following hepatic I/R injury, whereas DIZE pretreatment rescued this phenotype. Given IκBα inhibits NFκB translocation into the nucleus by masking the nuclear localization signals of NFκB proteins, and given hepatic I/R injury induces IκBα degradation thereby allowing unregulated translocation of NFκB to the nucleus, we speculated that DIZE suppresses NFκB-mediated proinflammatory response by preventing IκBα degradation. Indeed, there is evidence showing that DIZE treatment remarkably suppresses the NFκB signaling pathway and the production of proinflammatory cytokines in macrophages after LPS treatment^8^, so direct inhibition of proinflammatory response (NFκB activation and cytokines upregulation) in livers by DIZE may also contribute to protection of hepatic I/R injury. However, the detailed mechanism of effect of DIZE on NFκB activation during hepatic I/R injury remains to be defined in future studies.

As previously mentioned, it is believed that the formation of reactive oxygen species (ROS) and oxidative stress following reperfusion after prolonged ischemia are responsible for initiating detrimental cellular responses including inflammation and apoptotic/necrotic cell death, thereby leading to liver dysfunction and failure^23^. Among leukocytes, resident macrophages, known as Kupffer cells, and infiltrated neutrophils are major sources of ROS during hepatic I/R injury. Activated Kupffer cells and neutrophils generate superoxide through xanthine oxidase and/or NADPH oxidase, which are also present in sinusoidal endothelial cells and hepatocytes^24,25^; these are rapidly converted to hydrogen peroxide by SOD1 and SOD2 in the cytosol and mitochondria, respectively. In this study, we showed that H_2_O_2_ levels and 4-HNE expression (index of lipid peroxidation), and DNA oxidation significantly increased in the livers of mice subjected to hepatic I/R, and DIZE pretreatment significantly attenuated these effects. Furthermore, SOD1 levels decreased following hepatic I/R injury, and this decrease was prevented by DIZE pretreatment, indicating that DIZE reduces ROS generation and oxidative stress following hepatic I/R, thereby protecting against hepatic I/R injury. Interestingly, we also observed that levels of HO-1, an Nrf2-regulated survival factor that plays a critical role in the prevention of inflammation and oxidative stress^18,19^ were increased by DIZE pretreatment following hepatic I/R injury compared to pretreatment with vehicle. Upregulation of HO-1 is a pivotal cytoprotective mechanism that plays a key role in maintaining antioxidant/oxidant homeostasis during cellular injury, such as injury due to ischemia, hypoxia, hyperthermia, or radiation^26^. HO-1, a heat shock protein 32 (Hsp32), is an inducible HO isoform that converts heme into ferrous iron, biliverdin, and carbon monoxide, and numerous studies have demonstrated the therapeutic potential of HO-1 against hepatic I/R injury via its antioxidant, anti-inflammatory and anti-apoptotic properties^27–29^. Some studies have investigated the relationship between ACE2 and Nrf2 pathway-mediated HO-1 upregulation. Fang et al. reported that DIZE attenuates inflammatory response and oxidative stress by activating the Nrf2/HO-1/NQO1 pathway^30^ and Zhang et al. reported that the mRNA and protein expression of NQO1, another Nrf2 regulated gene, and HO-1 were increased by lentiviral-ACE2 treatment in human umbilical cord mesenchymal stem cells^31^.

Many studies have suggested that the protective effects of DIZE on diverse ischemic tissue injury, including the heart^7^, kidney^12^, and limb^32^ tissue injury, are mediated by ACE2 activation. We also agree that, at least in part, the protective effect of DIZE may be related to the Ang (1–7)/Mas receptor axis. However, in this study, we found that DIZE pretreatment increased ACE2 activity in plasma following hepatic I/R injury as evaluated by angiotensin 2 and angiotensin (1–7) levels, but DIZE pretreatment didn’t affect the changes of ACE2 activity in liver after sham or hepatic I/R injury. This result suggests that DIZE has a protective effect against hepatic I/R injury regardless of changes in ACE2 activity in the liver tissues, it can be considered as an indirect protective effect induced by an increase in angiotensin (1–7) in other major sources, heart, brain and kidney^33^. Indeed, activation of systemic inflammation such as circulating leukocytes and cytokines/chemokines is the primary pathophysiological mechanism mediating hepatic I/R injury so we suggest that the increase in angiotensin (1–7) in plasma from other tissues including kidney and heart by DIZE treatment is involved in the liver protection against I/R injury by inhibiting systemic inflammation. To clearly see whether the protective effect of DIZE on hepatic I/R injury is ACE2 dependent in liver or other organs such as heart, brain and kidney^33^, we need to confirm the DIZE-mediated liver protective effect against hepatic I/R in ACE2 knockout mice or ACE2 conditional knockout mice in the future study.

Taken together, although the underlying mechanism needs to be further defined, our data demonstrate that DIZE protects against ischemic liver injury by attenuating inflammation and oxidative damage, suggesting that DIZE may be a potential therapeutic agent for the prevention and treatment of ischemic liver failure. In addition, although, DIZE is not yet approved by US Food and Drug Administration (FDA) for treatment of human disease, our and other findings provide an opportunity to develop this drug for diverse diseases treatment such as kidney^12,14^, liver^6^, lung^5^ and heart^7^ diseases in the near future.

## Supplementary Information

Supplementary Figures.

## Data Availability

The datasets generated during and/or analyzed during the current study are available from the corresponding author on reasonable request.
