# Supplementary material for: Diminazene aceturate attenuates hepatic ischemia/reperfusion injury in mice
Source: Sci Rep. 2022 Oct 28;12:18158. doi: 10.1038/s41598-022-21865-2 (PMC9616812; doi:10.1038/s41598-022-21865-2)

Full unprocessed gel for Figure 2C

Bcl-2

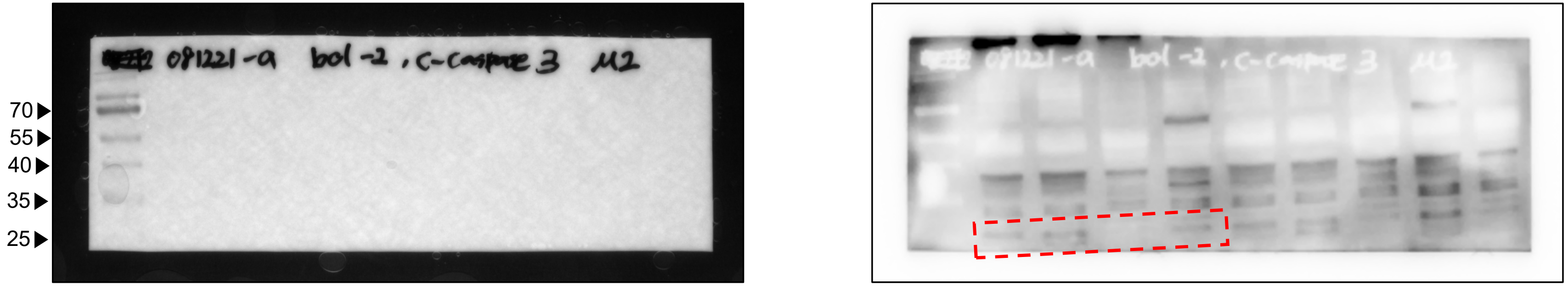

C-caspase 3

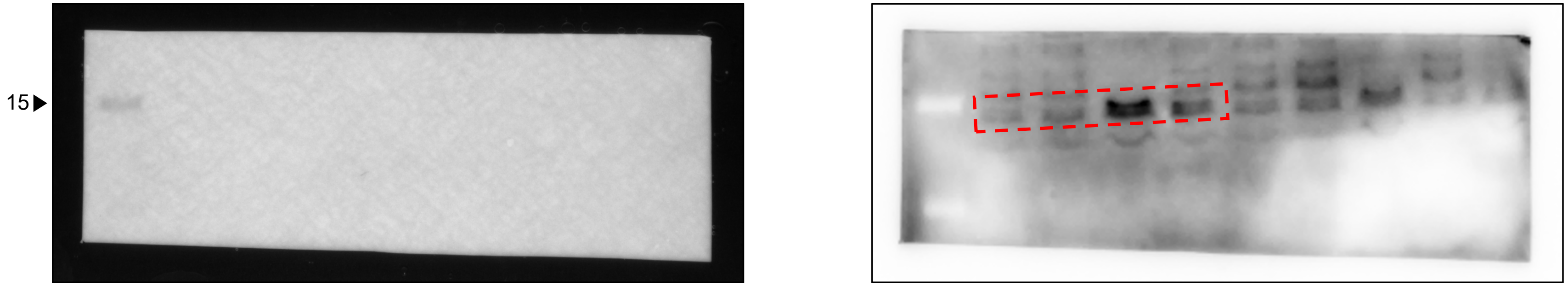

GAPDH

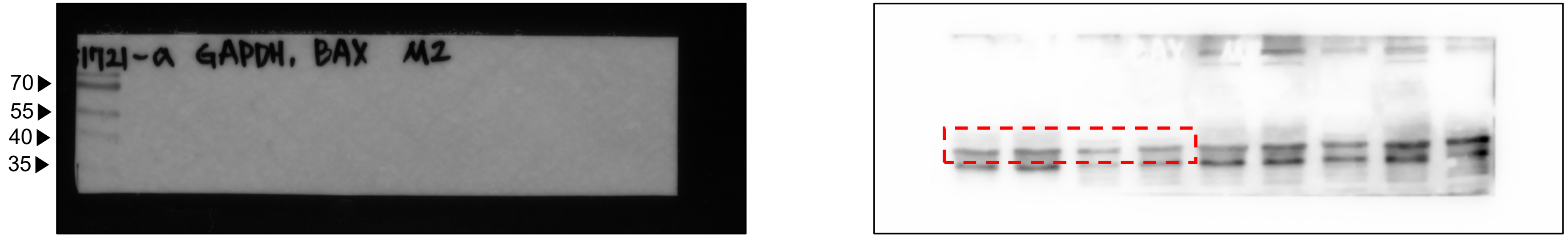

Full unprocessed gel for Figure 3C

Ly6G

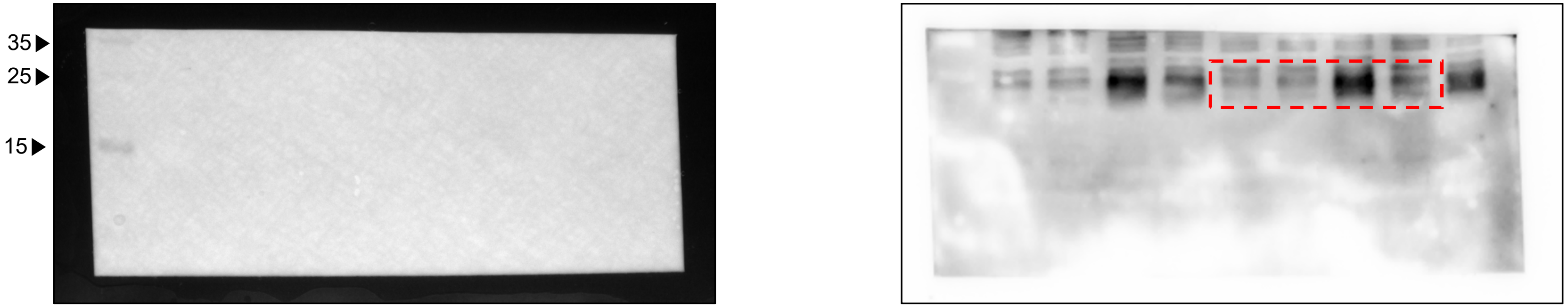

GAPDH

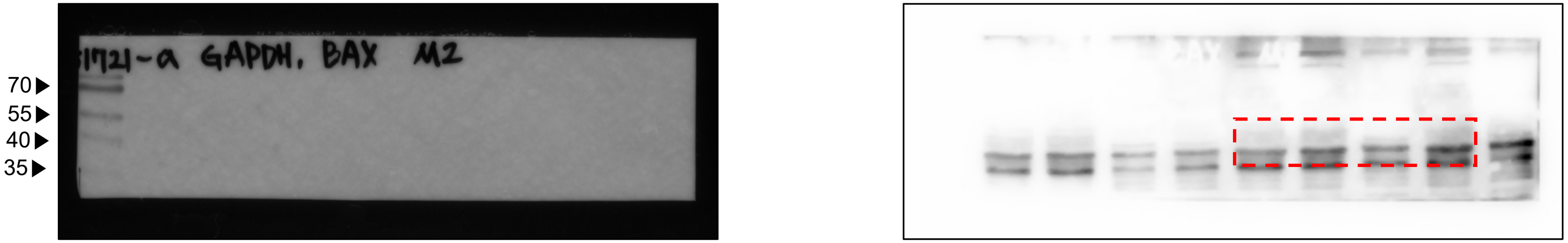

Full unprocessed gel for Figure 4G

NFκB

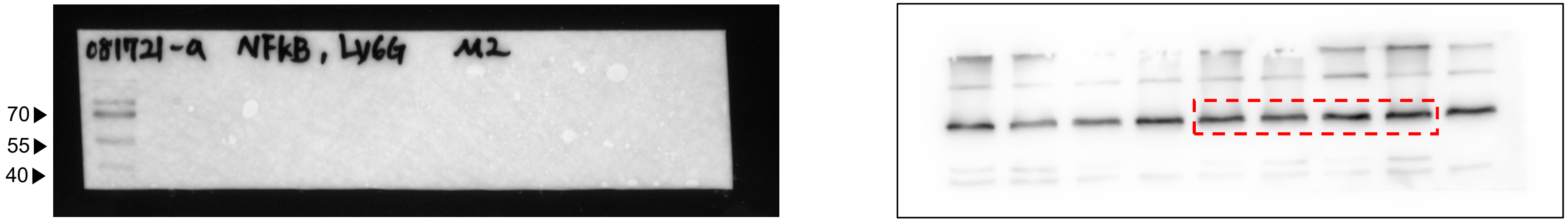

IκBα

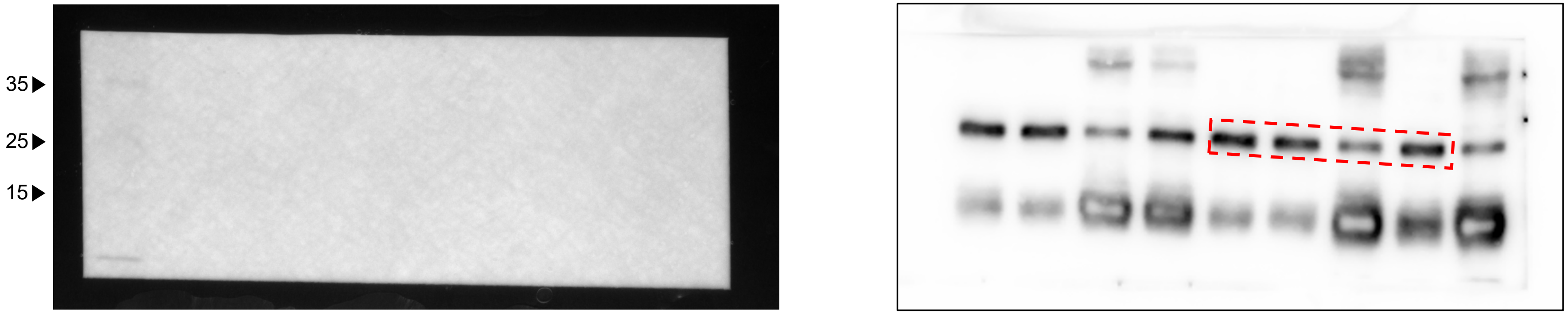

GAPDH

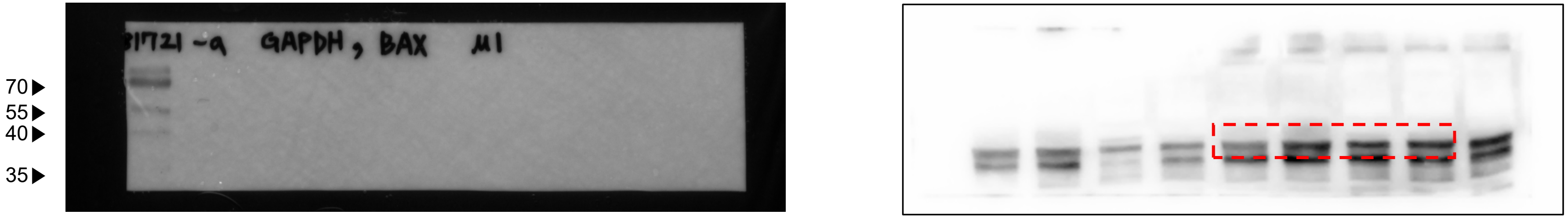

Full unprocessed gel for Figure 5B

Ly6G

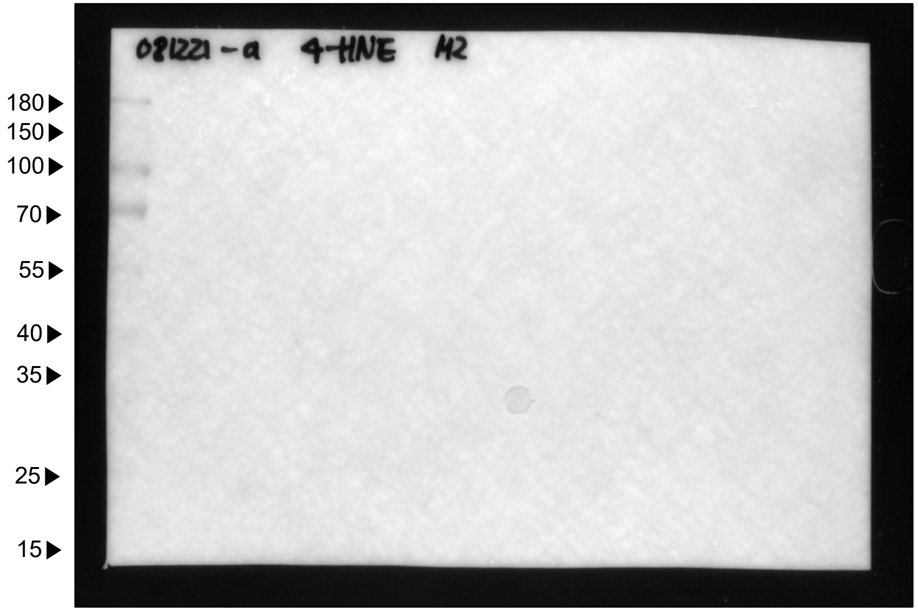

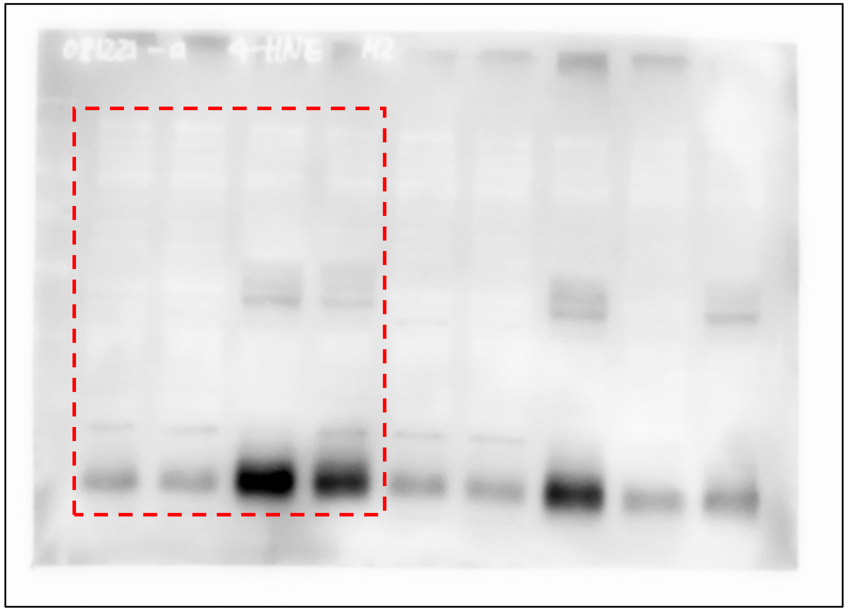

GAPDH

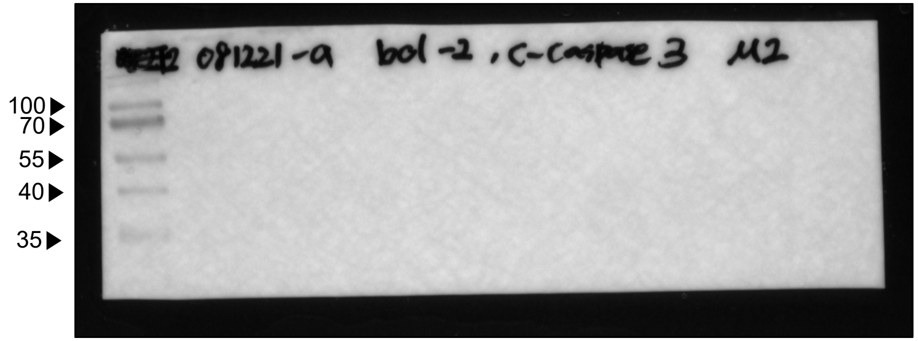

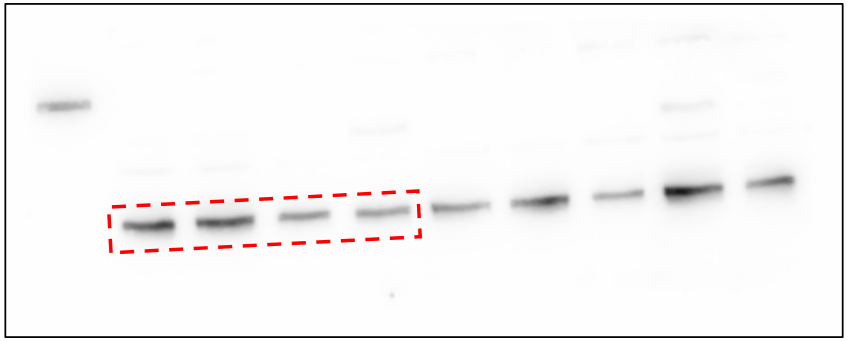

Full unprocessed gel for Figure 5F

HO-1

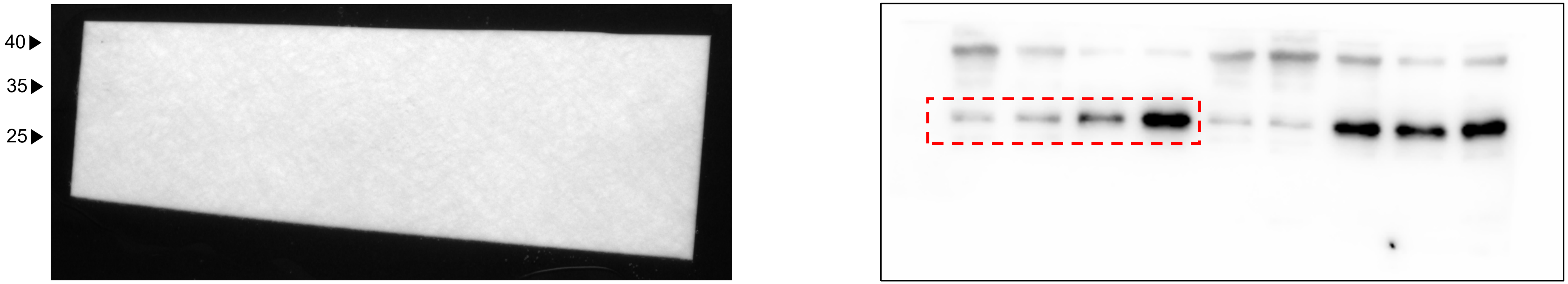

GAPDH

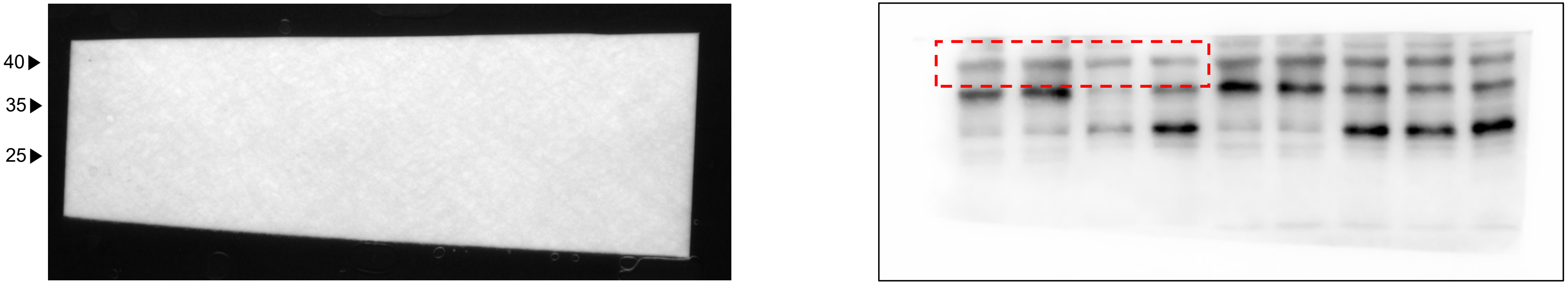

Full unprocessed gel for Figure 5F

SOD-1

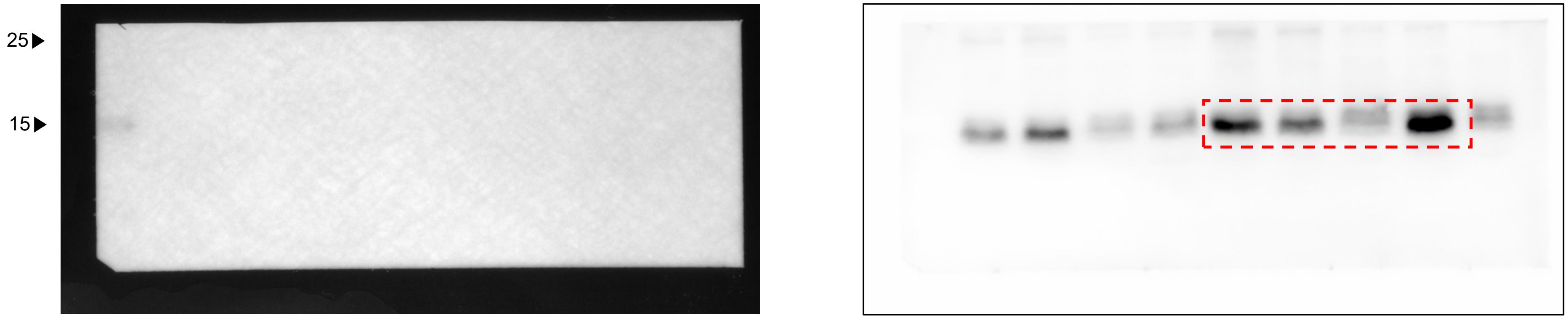

GAPDH

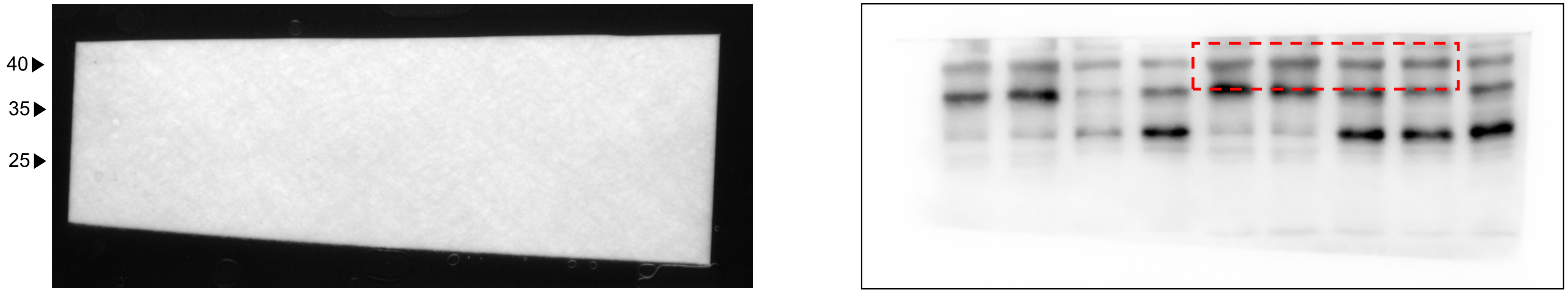

Supplement: Supplementary file 1 — Supplementary Figures. [file 41598_2022_21865_MOESM1_ESM.pdf]
